# The dimethadione-exposed rat fetus: an animal model for the prenatal ultrasound characterization of ventricular septal defect

**DOI:** 10.1186/s12872-023-03482-7

**Published:** 2023-09-09

**Authors:** Yiru Yang, GuoRong Lyu, Shaozheng He, Hainan Yang, Shangqing Li

**Affiliations:** 1 The Second Clinical Medical College of Fujian Medical University, Fujian, China; 2 Collaborative Innovation Center for Maternal and Infant Health Service Application Technology, Quanzhou Medical College, Quanzhou, China

**Keywords:** Ventricular septal defect, Animal model, Prenatal ultrasonic diagnosis, Pathology

## Abstract

**Background:**

Ventricular septal defect (VSD) is the most prevalent congenital heart disease (CHD) and is easily misdiagnosed or missed. An appropriate VSD animal model could be used to analyze the ultrasound characteristics and their related pathological bases, and provides the opportunity to further explore the pathogenesis of VSD. Currently, little is known about whether ultrahigh-frequency ultrasound biomicroscopy (UBM) is suitable to diagnose VSD of fetal rats. There is no research on whether a dimethadione (DMO)-induced fetal VSD model is suitable for the observation and analysis of imaging characteristics and the associated pathological basis.

**Methods:**

We used DMO to induce VSD. UBM was used to perform the prenatal ultrasound characterization. With the pathological results used as the gold standard, the ultrasound characteristics and their related pathological bases were analyzed.

**Results:**

The incidence of VSD in the DMO group was 42.05% and 39.71% (diagnosed by UBM and pathology, respectively, *P* > 0.05). The prenatal ultrasound findings and pathological basis of various diseases, including isolated VSD, complex CHD containing VSD, and extracardiac lesions, were detected and discussed. It was discovered that some fetuses showed features of noncompacted ventricular myocardium, and for the first time, clusters of red blood cell traversing the cardiomyocytes.

**Conclusions:**

The DMO-induced VSD model is a low-cost model with a high success rate and is suitable for the observation and analysis of VSD. UBM is suitable for evaluating VSD.

## Introduction

Ventricular septal defect (VSD) is the most prevalent congenital heart disease (CHD) and is easily misdiagnosed or missed [1–4]. It can exist alone (hereafter referred to as an isolated VSD) or as a component of another cardiac abnormality, such as tetralogy of Fallot, double outlet right ventricle or persistent truncus arteriosus (hereafter referred to as a complex CHD containing VSD) [1, 2]. It has been confirmed that developments on echocardiography have greatly improved the detection of isolated VSD [5]. An appropriate VSD animal model could be used to analyze the ultrasound characteristics and their related pathological bases, and provides the opportunity to further explore the pathogenesis of VSD. Combining the ultrasound and pathology features is helpful to provide novel ideas for accurate prenatal diagnosis of VSD. Currently, little is known about whether ultrahigh-frequency ultrasound biomicroscopy (UBM) is suitable to diagnose VSD of fetal rats.

Previous studies have demonstrated that exposure to the anticonvulsant trimethadione during pregnancy can lead to fetal trimethadione syndrome, which manifests as clinical features comprising cardiac malformations and intrauterine growth retardation [6]. Dimethadione (DMO), the N-demethylated metabolite of trimethadione, resembles the teratogen and active metabolite of trimethadione [7]. With low maternal and fetal toxicity, DMO induces VSD at a rate of 65–74% during the key window of heart development in rats (from 8.5 days to 11 days of pregnancy) [8, 9], while the natural incidence of cardiovascular malformations in Sprague-Dawley (SD) rats ranges only from 0.02 to 0.72% [10]. Besides, the success rate of DMO for inducing fetal VSD is significantly higher than that of sodium arsenic, homocysteine and VLA-4 antagonist derivatives [11–14]. Purssell et al. [9]identified the optimal conditions for use of the UBM and proved that UBM could identify VSD in fetuses. Currently, there is no research on whether a DMO-induced fetal VSD model is suitable for the observation and analysis of imaging characteristics and the associated pathological basis. Thus, we speculated that VSD induced by DMO would be an excellent animal model for detecting characteristics of VSD by prenatal ultrasound via UBM. To address this, our study used a DMO-induced VSD animal model combined with UBM for prenatal ultrasound and pathology, and explored the feasibility of studying the structural and hemodynamic changes of fetal VSD with this model to lay the foundation for studying the pathogenesis and identifying features of VSD, so as to enable an accurate diagnosis of VSD.

## Materials and methods

### Establishment of the animal model

SD rats were purchased from Shanghai SLAC Laboratory Animal Co., Ltd. Female and male SD rats in estrus (11 ~ 23 weeks-old) were kept in cages at a ratio of 2:1 for 12 h. Female rats with vaginal plugs were immediately separated from male rats, and the day was recorded as embryonic day 0 (D0). According to “resource equation” method, a total of 16 pregnant rats was included [15]. The dams were randomized to a control group and a DMO group (simple randomization). The DMO group was given 300 mg/kg DMO (drug concentration, 60 mg/ml) by oral gavage at 19:00 on D8 once every 12 h for a total of 6 times (the cumulative dose was 1800 mg/kg). The control group was given the same dose (5 ml/kg) of distilled water at the same time. The rats were fed standard food and distilled water ad libitum and received humane care. This study was approved by the Medical Ethics Committee of the Second Clinical Medical College of Fujian Medical University (2021-73). Without exception, all dams survived until the end of the experiment, with no signs of illness such as weight loss, increased temperature, fur changes, or trembling.

### Prenatal ultrasound

At D18, dams were injected intraperitoneally with pentobarbital (40 mg/kg) and scanned by Vevo 2100 ultrahigh-frequency ultrasound biomicroscopy (transducer frequency, 24 ~ 40 MHz). Ultrasound was performed by investigators blinded to the treatment. The images were obtained by means of 3 modes: brightness mode (B-mode) was used to observe the chambers and ventricular septum of the fetal heart; color Doppler mode was used to view the presence of transseptal blood flow; and pulse-wave Doppler mode was used to detect bidirectional blood flow supporting the diagnosis of VSD.

At the beginning of the scan, the number of litters and the location of the fetus were confirmed. With the maternal bladder used as an anatomical reference point, fetuses were labelled based on the distance from the cervix. From near to far, the fetus on the left was labelled as L1, 2, 3, etc., and the right was labelled as R1, 2, 3, etc. Sequentially, the heart of the fetus was scanned, and a preliminary diagnosis of CHD was made.

### Pathology

The dams were euthanized following ultrasound, and the caesarean section was performed right after euthanasia. Microdissection was performed to observe the position and connection of the heart and great vessels. Subsequently the heart was removed and washed in cold PBS solution. The heart was soaked in 10% formalin solution for at least 2 h and then dehydrated in gradient ethanol solution overnight. The heart tissue was embedded in paraffin, and the wax block was cut into 3-µm slices. HE staining was performed in accordance with standard protocols by researchers blinded to the treatment.

### Statistical method

SPSS 20.0 software (SPSS Corporation, Chicago, USA) was used for statistical analysis in accordance with the Brief Guide. Normal data are expressed as the mean values ± standard deviation (mean ± SD). Skewed data are presented as the median, lower quartile and upper quartile. The differences between two groups were compared by means of the rank-sum test, *t*-test or chi squared test. *P* < 0.05 was considered statistically significant.

## Results

A total of 16 dams were included. The general situations of the dams and fetuses in the two groups are shown in Table 1. There was no significant difference in age, weight, number of implanted embryos, or abortions among the dams between the control group and the DMO group. The weight of the dams in the DMO group increased by 88.88 ± 28.51 g during pregnancy, and the average number of viable fetuses was 11 ± 5.24 per litter, which was slightly lower than that in the control group without statistical significance (both *P* > 0.05). Additionally, the weight of viable fetuses was significantly lower in the DMO group than in the control group (*P* < 0.01).

**Table 1:** The influence of DMO on dams and fetuses

	Control	DMO	P
Dams			
Number (n)	8	8	\
Age (weeks)	16.13 ± 4.55	15.88 ± 4.05	0.91
Weight (g)	288.50 ± 39.03	279.25 ± 38.89	0.64
Changes of weight (g)	102.88 ± 45.61	88.88 ± 28.51	0.47
Implantations (n)	14.75 ± 2.49	13.25 ± 3.33	0.33
Viable fetuses with normal appearance (n)	14.38 ± 2.83	11.00 ± 5.24	0.31
Early abortions (n)	0.00 (0.00, 0.00)	0.00 (0.00, 0.75)	0.49
Late abortions (n)	0.00 (0.00, 0.75)	1.00 (0.00, 1.00)	0.12
**Fetuses**			
Body weight (g)^*^	2.41(1.75, 2.63)	1.75(1.11, 2.06)	< 0.01^*^

DMO, dimethadione; *P < 0.05

Fetuses with abortions or subcutaneous edema were excluded. In the DMO group, a total of 88 fetuses with a normal appearance were included. Limited by the relatively insufficient penetration of UBM, it is difficult to perform heart scans on fetuses lying deep in the abdominal cavity. Thus, the number of fetuses scanned is less than that of viable fetuses. The incidence of VSD diagnosed by UBM was 39.71% (27/68), and the incidence of VSD confirmed by pathology was 42.05% (37/88). The statistical difference was insignificant (Table 2, *P* > 0.05).

**Table 2:** The comparison of heart defects detection rate diagnosed by ultrasound and pathology

	Negative [n(%)]	Positive [n(%)]
**Ventricular septal defect** ^a^		
UBM	41(60.29%)	27(39.71%)
Pathology	51(57.95%)	37(42.05%)
**Persistent truncus arteriosus** ^a^		
UBM	65(95.59%)	3(4.41%)
pathology	83(94.32%)	5(5.68%)
**Noncompacted ventricular myocardium** ^a^		
UBM	58(85.29%)	10(14.71%)
pathology	72(81.82%)	16(18.18%)

UBM, ultrasound biomicroscopy; ^a^, P > 0.05

**Fig. 1:**
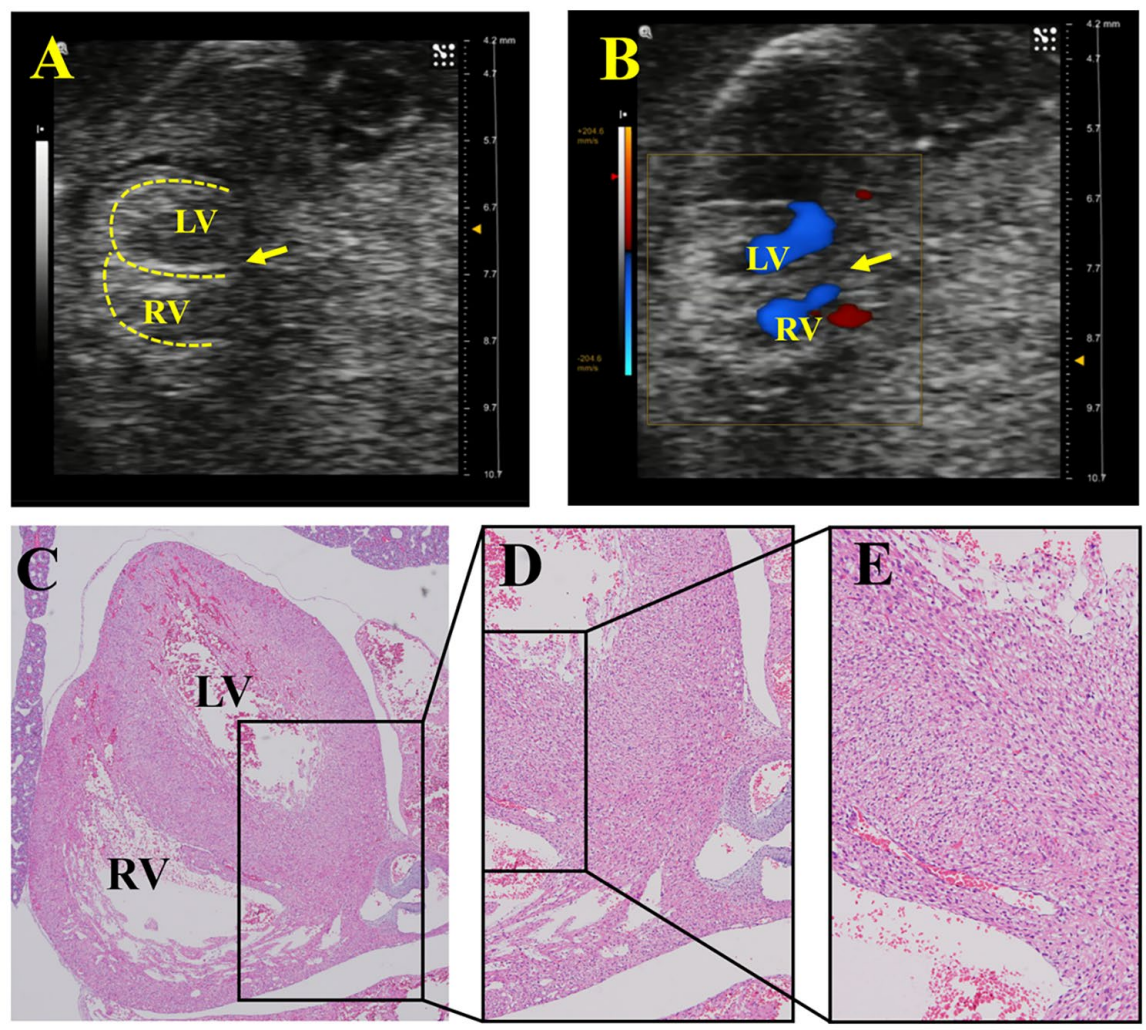
**Prenatal cardiac ultrasound and pathological images of normal fetuses. A** is a B-mode image, where the echoes of the ventricular wall and septum can be observed. **B** is a color Doppler image, and no transseptal blood flow was observed. **C**, **D**, and **E** are all pathological images (magnification are 40×, 100× and 200×, respectively), with an intact ventricular septum and tightly arranged cardiomyocytes of the ventricular septum. (The dotted line indicates the ventricular wall and septum, and the arrow points to the ventricular septum. LV, left ventricle; RV, right ventricle)

**Fig. 2:**
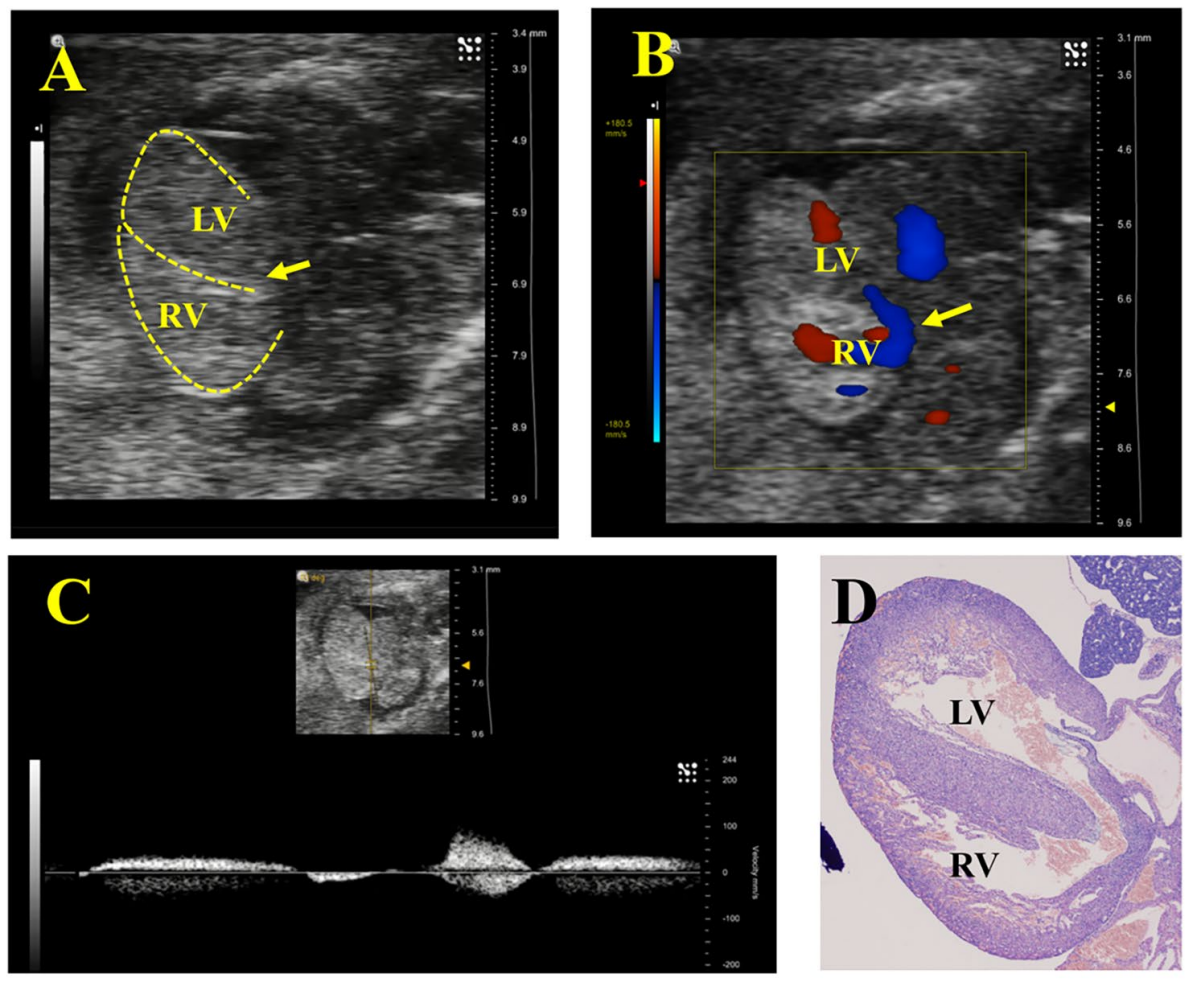
**Prenatal cardiac ultrasound and pathological images of ventricular septal defect (VSD) fetuses. A** is a B-mode image. **B** is a color Doppler image, showing transseptal blood flow. **C** is a pulse-wave Doppler mode, with detectable bidirectional blood flow at the ventricular septum. **D** is a pathological image (magnification is 40×), confirming a VSD. (The dotted line indicates the ventricular wall and septum, and the arrow points to VSD. LV, left ventricle; RV, right ventricle)

**Fig. 3:**
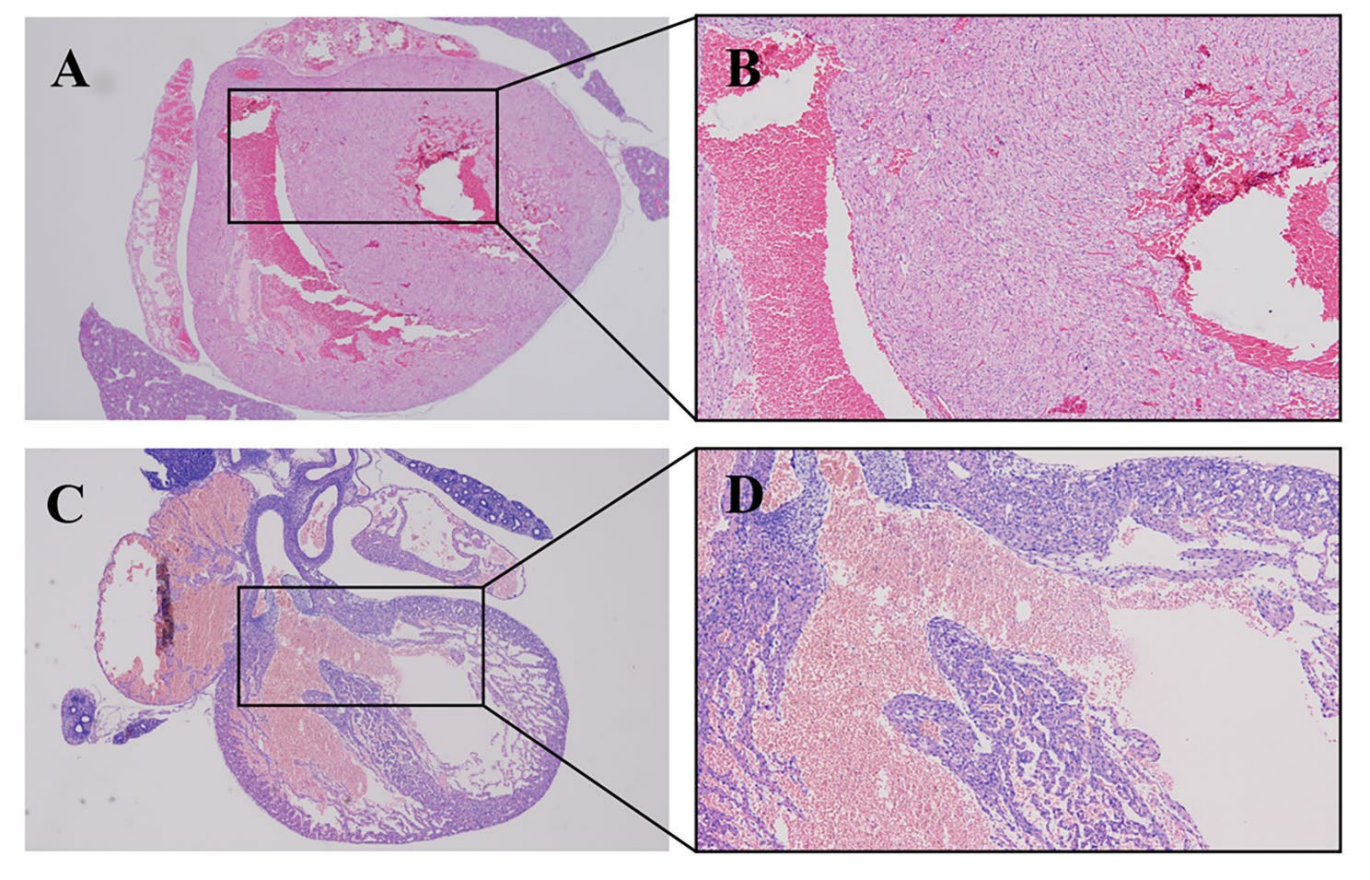
**Comparison of fetal heart pathological images. A** and **B** are pathological images of fetal hearts in the control group (magnifications are 40× and 100×, respectively), showing complete ventricular septa and tightly arranged cardiomyocytes. **C** and **D** are pathological images of VSD fetal hearts (magnifications are 40× and 100×, respectively), indicating thin myocardia and an interrupted ventricular septum with a “bifurcated” shape

UBM could be used to observe most of the fetal heart chambers and septa through the abdomen of the dam, to make a preliminary judgement of VSD, which was then proven by pathology (Figs. 1 and 2). Although the B-mode can recognize the ventricular wall and septum through the high echo of the myocardium, the appearance of a fetal heart with a tiny VSD is similar to that of a normal heart on two-dimensional ultrasound and thus has a high likelihood of missed diagnosis. Combined with the transseptal blood flow in color Doppler mode and bidirectional blood flow in pulse-wave Doppler mode at the defect, VSD can be diagnosed more accurately. As shown in Fig. 3, the wall and septal cells of fetal rats in the control group were tightly arranged, while the cardiomyocytes of the VSD fetus were loose and more likely to present a “bifurcated” ventricular septum.

**Fig. 4:**
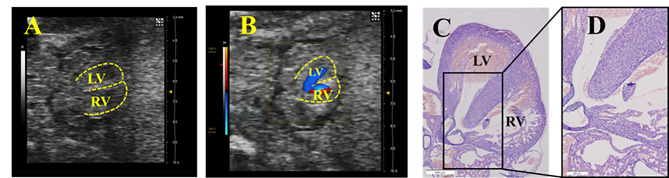
**Prenatal ultrasound and pathological images of fetal rats with persistent truncus arteriosus. A** is a B-mode image. **B** is a color Doppler image, showing blood flow from the left and right ventricles going through the defect of the septum and into the arterial trunk together. **C** and **D** are pathological images (magnifications are 40× and 100×, respectively), showing that the left and right ventricular chambers share arterial valves, leading to the common trunk. (The dotted line indicates the ventricular wall and septum. LV, left ventricle; RV, right ventricle)

In the DMO group, there were not only isolated VSDs but also complex CHDs containing VSDs, namely, persistent truncus arteriosus. A total of 3 cases (4.41%) of persistent truncus arteriosus were found on prenatal ultrasound. In color Doppler mode, blood flow from the left and right ventricles went through the VSD and into the common arterial trunk. Pathology revealed that 5 cases (5.68%) shared one set of arterial valves for the left and right ventricular chambers, leading to a common trunk (Fig. 4). There was no statistically significant difference between the two methods (Table 2).

**Fig. 5:**
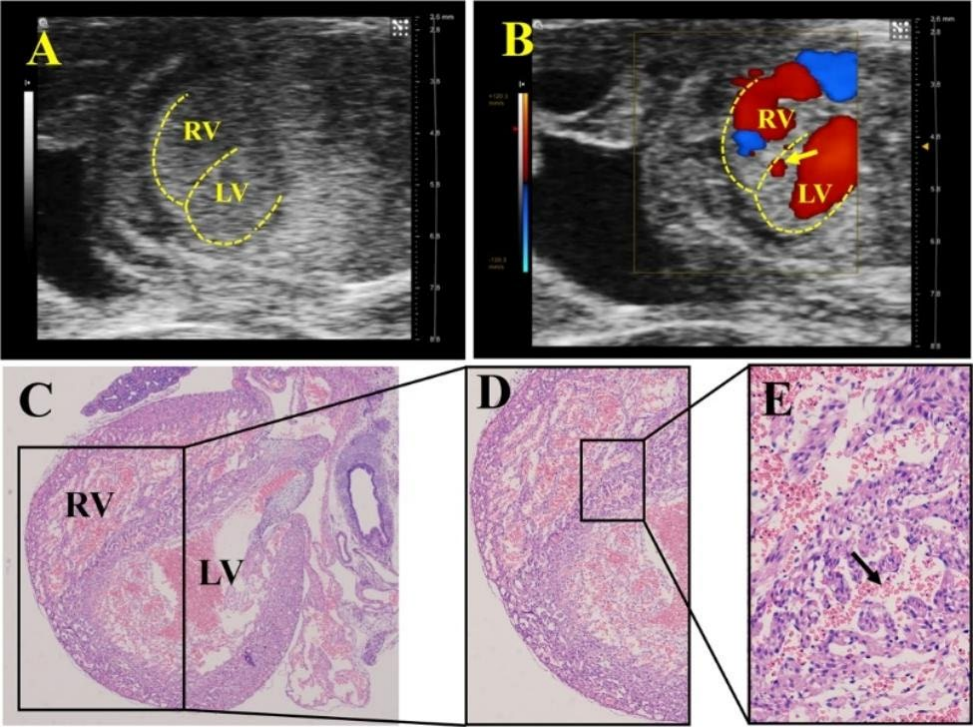
**Prenatal ultrasound and pathological images of fetuses with a “spongy” myocardium. A** is a B-mode image; **B** is a color Doppler image, revealing that a small amount of blood flow signals appears in the septum (arrow). **C**, **D**, and **E** are all pathological images (magnifications are 40×, 100×, and 400×, respectively). The ventricular septum was roughly intact, but the myocardium was loosely arranged with RBCs crossover (arrow). (The dotted line indicates the ventricular wall and septum. LV, left ventricle; RV, right ventricle)

In contrast to the typical VSD, a kind of “spongy” myocardium appeared in DMO-induced fetuses. Ten cases (14.71%) were found by UBM, and 16 cases (18.18%) were confirmed by pathology (Table 2, *P* > 0.05). There was no clear interruption of the ventricular septum in such cases, but a significantly reduced echo of the myocardium, a loose cell arrangement and a small amount of blood flow through the ventricular septum were detected (Fig. 5). Compared with the control group, the predominant characteristics of prenatal ultrasound in the DMO group were the lower echo of the ventricular wall and septum, the unclear distinction between the myocardium and surrounding tissues in B-mode, and a small amount of low-velocity blood flow passing through the ventricular septum in color Doppler mode. Pathological images showed that the cells in the inner layer were arranged loosely with lots of ventricular trabeculae dizzying arrayed and the intertrabecular communicated with the ventricular cavity, representing the features of noncompacted ventricular myocardium (NCVM). The ventricular septum was roughly intact, while the cells of ventricular septum were arranged in a “spongy” way, with clusters of red blood cells (RBCs) traversing the cardiomyocytes, showing obvious differences from the regularly and closely arranged myocardium in the control group.

**Fig. 6:**
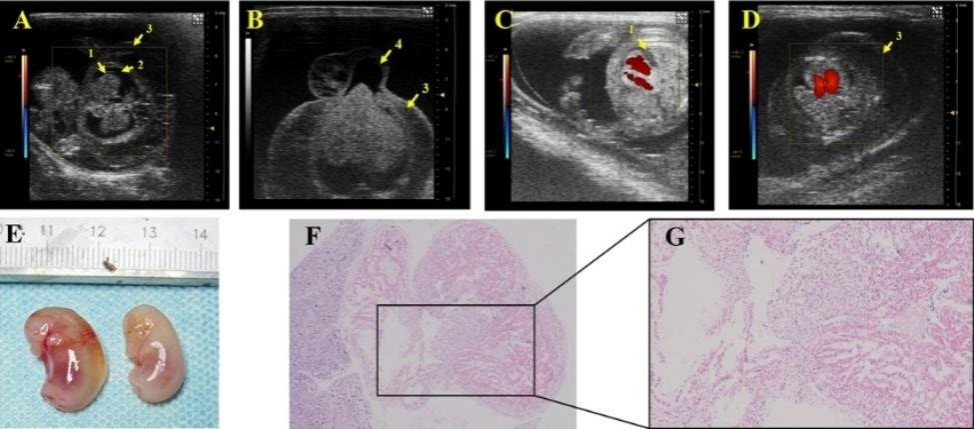
**Ultrasonic, anatomical and pathological manifestations of extracardiac lesions. A** is a color Doppler image of an aborted fetus with pericardial effusion, pleural effusion and subcutaneous edema. **B** is a B-mode image of a fetus with umbilical hernia and subcutaneous edema. **C** is a color Doppler image of a viable fetus with pleural effusion. **D** is a color Doppler image of a viable fetus with subcutaneous edema. **E** is the comparison of a fetus with subcutaneous edema (left) to a fetus with a normal appearance from the same litter (right). **F **and **G** are pathology images of the fetus with edema (magnifications are 40× and 100×, respectively), indicating VSD, loose heart tissue, and an incompletely formed heart chamber. (Arrow 1 indicates pericardial effusion, arrow 2 indicates pleural effusion, arrow 3 indicates subcutaneous edema, and arrow 4 indicates umbilical hernia.)

In addition to the abovementioned malformations of the cardiovascular system, pericardial effusion, pleural effusion, subcutaneous edema, and umbilical hernia were also found in the DMO group (Fig. 6). The fetus in Fig. 6-A has a preliminarily formed heart and lung structure, but the blood flow signal of the heart cannot be detected in color Doppler mode, indicating an aborted fetus with pericardial effusion, pleural effusion and subcutaneous edema. The fetus in Fig. 6-B has an umbilical hernia and subcutaneous edema. Figure 6-C and -D show a surviving fetus that also suffers from pleural effusion and subcutaneous edema. Figure 6-F and -G are pathological images of a fetus with edema, indicating VSD, loose heart tissue, and an incompletely formed heart chamber.

## Discussion

In our study, fetal VSD models, including isolated VSDs and complex CHDs containing VSDs, were successfully generated via DMO. The prenatal ultrasound characteristics of VSD and some extracardiac lesions were observed by UBM and confirmed by pathology. Our results revealed that exposure of fetuses to DMO can successfully induce VSD without affecting their survival rate or that of the dams. This model can be used for observation and analysis of the prenatal cardiac ultrasound characteristics and pathological basis of VSD, contribute to finding the imaging features for precise prenatal diagnosis, and provide novel clues for precise diagnoses.

In the field of embryonic cardiac defects, animal models that are suitable for large-scale observation of anatomical and pathological features via imaging are exceedingly rare. Gene knockout models are technically demanding and expensive [16]. The imaging assessments of surgically generated shunt models are generally influenced by hyperplasia and scars. In addition, this kind of model is difficult to clarify whether the occurrence order of cardiac abnormalities and hemodynamic changes are the same as spontaneous CHD [17]. Pluripotent stem cells are not mature enough to simulate the native heart tissue or structure and cannot be used to assess imaging features [18]. Teratogens, one of the etiologies of human CHD, can be used to generate animal models that are cost effective and close to the clinical situations [19]. Thus, in our study, a teratogen was used to construct an animal model.

A variety of teratogens have been employed to construct animal models of CHD. VLA-4 antagonist derivatives could induce VSD. The incidence rates in rats and rabbits are 48% and 50%, but the related fetal mortality rates are also up to 24.9% and 55.6%, respectively [14]. Sodium arsenic may disrupt the expression of myocardium-related genes and inducing CHD [11, 12]. Zinc deficiency may cause heart malformations through high levels of Cx43 [20]. However, the incidences of CHD with the above two methods are less than 40% [11, 12, 20]. DMO has teratogenicity related to arrhythmia, hypoxia and ischemia-reperfusion injury [21]. In our study, 42.05% of fetuses in the DMO group developed VSD. Compared with other CHD-related teratogens, DMO has no significant maternal or fetal toxicity, leading to a higher incidence of VSD, which makes DMO a more ideal teratogen.

The incidence of DMO-induced VSD in this study was 42.05%, which is somewhat different from the previously reported 65%~74% [8, 9]. The possible reason is that some fetuses with edema have poor cardiac development, which is not conducive to supersonic observation. Therefore, this kind of situation was not included in the statistics. The development of the murine heart is similar to that of humans. Development starts at D8, and a linear myocardial heart tube forms at D8.5. Then, the chamber begins to form [22, 23]. 98% of normal rat fetuses have a completely developed ventricular septum after D16 [9]. In our study, the time of dissection approached the delivery time. Nevertheless, the ventricular chamber of some edematous fetuses was still not fully formed and could not contain as many RBCs as normal developed fetuses, and the related hemodynamic changes were not conducive to ultrasound observation. Thus, such cases were excluded from the statistics.

Previous studies used ultrasound to observe the heart structure and function of CHD rats [24–26]. The rat fetus, by contrast, has a smaller heart and lower blood volume, which require higher resolution ultrasound equipment. In addition, the scanning angle is limited by factors such as multiple pregnancy, fetal position and the movement of the dam’s uterus. Consequently, it is more difficult to perform prenatal ultrasound of the fetal heart. It was reported that the Vevo2100 system has excellent diagnostic capabilities for fetal CHD [27]. A previous study found VSD, persistent truncus arteriosus, and cardiomyopathy in DMO-induced fetuses [9]. Our study not only successfully confirmed the ultrasound characteristics of abovementioned diseases by the Vevo2100 system, but also observed the blood flow features of the persistent truncus arteriosus in color Doppler mode and pathology, providing new diagnostic clues of rat VSD.

Some fetuses was observed a “spongy” myocardium, numerous ventricular trabeculae and the intertrabecular communicated with the ventricular cavity, which was exactly the characteristics of NCVM [28]. NCVM ordinarily influenced the left ventricle rather than ventricular septum in previous clinical research [29]. We found that the ventricular septum could also be “spongy”, and discovered clusters of RBCs in the “spongy” ventricular septum. The ventricular septum was not interrupted, while there might be a mixing of oxygen-enriched blood and hypoxic blood. Further research is needed to clarify whether this lesion can result in secondary changes that are similar to VSD and NCVM. Additionally, we discovered lesions including multiple serous effusions, subcutaneous edema, and umbilical hernia, supplying ultrasound and pathological basis for integrated analysis of fetal VSD and its comorbidities.

This study has several limitations. Henderson et al. [30] showed that during the gestation period of approximately 20 days in mice, the fetal heart did not complete development until approximately 18 days of gestation. Besides, Purssell et al. [9] also confirmed that 98% of the rats were completely closed after 16 days of gestation. Murine form ventricular septum in the middle and late stages of pregnancy, so this model has limited value in early pregnancy. Because of the relatively insufficient penetration of UBM, fetuses lying deep in the abdominal cavity did not have heart scans. Although a “spongy” ventricular septum has been observed, the mechanism of its formation still needs to be explored. This animal model is induced by a teratogen, and whether some of the identified lesions are universal remains to be evaluated.

## Conclusions

The DMO-induced rat VSD model is convenient to operate and has a high success rate. It is an animal model that is suitable for observation and comparative analysis of the prenatal ultrasound characteristics of VSD. Given the relevant pathological basis of this model, it is expected to provide new clues for carrying out accurate diagnoses of VSD by prenatal ultrasound and for identifying novel diagnostic biomarkers of VSD with molecular biological or high-throughput technologies.

## Data Availability

The datasets used or analysed during the current study are available from the corresponding author on reasonable request.

## Abbreviations

VSD: ventricular septal defect
CHD: congenital heart disease
UBM: ultrasound biomicroscopy
DMO: dimethadione
SD: Sprague-Dawley
NCVM: noncompacted ventricular myocardium
RBCs: red blood cells
